# Unusual Presentation of Kawasaki Disease in a 13-year-old Saudi Boy

**DOI:** 10.7759/cureus.4053

**Published:** 2019-02-12

**Authors:** Rola Sleiman, Tahani Almohayya, Hussam Al Hennawi

**Affiliations:** 1 Department of Pediatrics, Dr. Sulaiman Al Habib Medical Center, Ryadh, SAU; 2 Department of Pediatrics, Dr. Sulaiman Al Habib Medical Center, Riyadh , SAU; 3 Department of Pediatrics, Dr. Sulaiman Al Habib Medical Center, Riyadh, SAU

**Keywords:** kawasaki disease, kd, vasculitis, saudi arabia

## Abstract

Kawasaki disease (KD), formerly known as mucocutaneous lymph node syndrome, is one of the most quintessential self-limiting forms of vasculitides among children. Nevertheless, sparse cases of adulthood KD have been also identified. Despite the self-limited (indolent) nature of this disease, patients tend to present with fever and signs of acute inflammation which may averagely last for up to 12 days without treatment, yet high index of clinical suspension is needed in atypical cases early during the course of the disease in order to minimize associated morbidity and mortality. Herein, we report an unorthodox case of KD of a 13-year-old male patient who started with cervical lymph node enlargement, followed by fever.

## Introduction

Kawasaki disease (KD), first identified and described in Japan by Dr. Tomisaku Kawasaki in 1976, is an acute inflammatory self-limiting systemic illness predominately affecting medium-sized muscular arteries of unknown cause, which typically lasts for an average period of 12 days without treatment [1-3]. KD is emerging as one of the prime causes of acquired heart disease, giving rise to coronary artery aneurysm as the most feared sequela among the pediatric population [4-5]. Moreover, incidents of KD most commonly occur between the age group of six months to five years, with exceedingly rare occurrence in patients over nine years of age and above accounting for <1% among 8,000 diagnosed KD patients surveyed in Japan and 7.4% in a retrospective study in Canada [6-7] with a slight male to female predominance of 1.5:1 [8]. KD is a worldwide disease prevalent among the Japanese population and other Asian countries [9]. With regard to Saudi Arabia, quite extensive experience with KD involving central, western, and eastern provinces has been documented sparsely, with a reported incidence of 7.4 per 100,000 children below five years of age in a retrospective study in the eastern region [10-11]. However, true overall incidence cannot be determined at the moment due to the lack of comparable studies [10]. Of note, no laboratory tests or other means of investigations have yet been developed for diagnosing KD. However, diagnosing KD can be established with reference to international criteria taking into consideration a patient's clinical presentation [9]. With that being said, atypical conundrums of KD may arise and indeed atypical cases are on the rise hindering proper diagnosis and management [2]. To our knowledge, this is the first reported case of KD in a 13-year-old male in KSA.

## Case presentation

**Figure 1:**
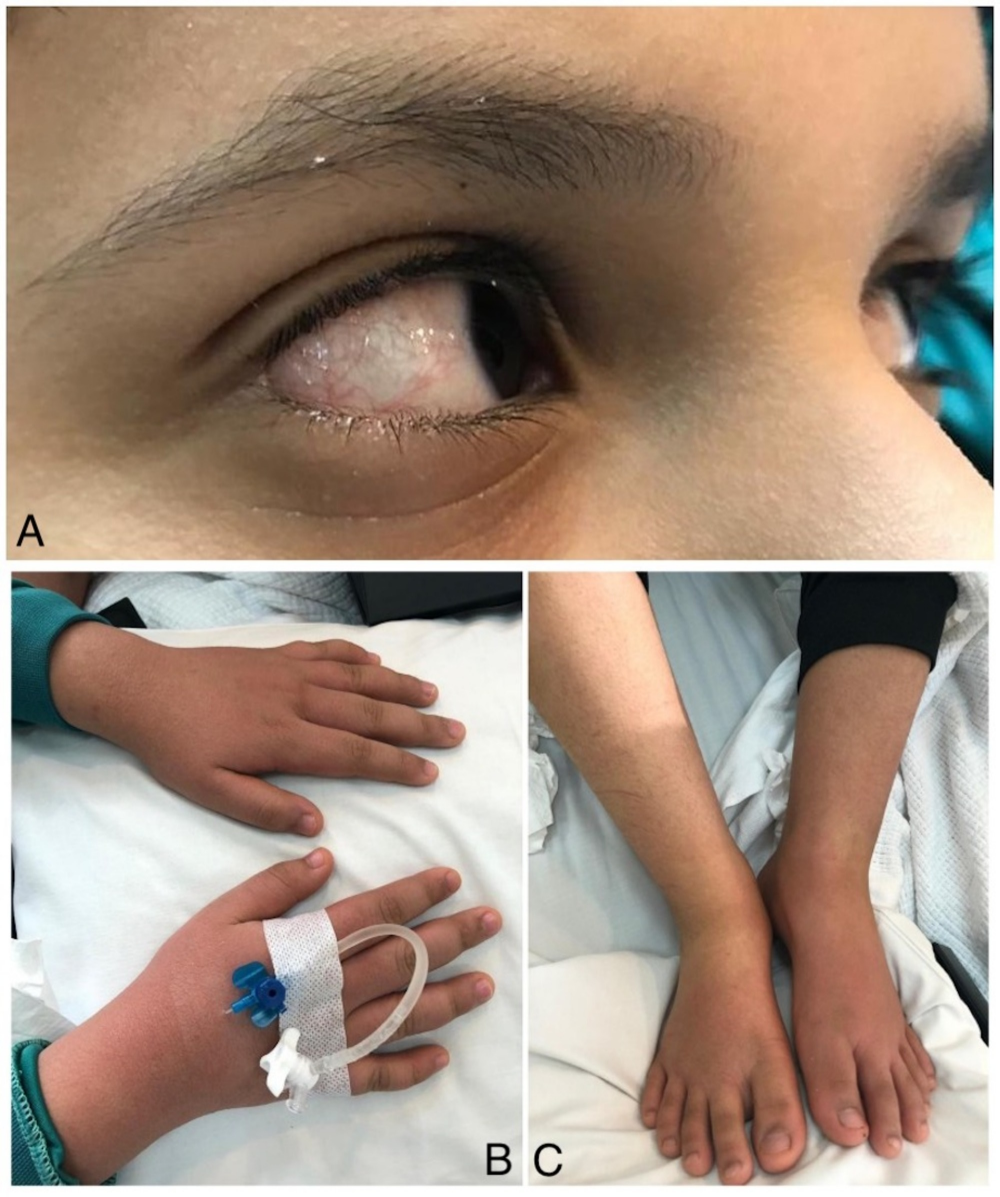
Symptoms observed during initial hospital visits Acute non-purulent bilateral conjunctivitis (A); bilateral upper and lower extremity edema (B, C)

The chronological history of an otherwise healthy 13-year-old male patient started when he first appeared in the clinic complaining of a sore throat and a sand-paper like rash involving both hands for one day. Physical examination revealed mildly congested erythematous pharynx and anterior right non-exudative cervical lymph node enlargement of approximately 5 x 3 cm^2^. The neck mass was firm and movable, tender to touch, and best visualized when the patient asked to move his head to the opposite side. Symmetrical papular rash involving both hands with an equal distribution was also noticed. No other abnormal physical examination findings were recognized at this initial stage. For the aforementioned findings, the patient was suspected of having scarlet fever for which strep throat test was ordered and turned back negative. The patient was then instructed to report back if there are signs of fever along with close monitoring of cervical lymph node enlargement by the parents and was discharged on an anti-histamine (Cetirizine) for itching. Two days later, the patient came back with high-grade fever, bilateral conjunctivitis (Figure 1A), bilateral hands, and feet edema (Figure 1B, 1C) with increased itching and appearance of macular rash over his body. At this stage, blood investigations revealed the following: hemoglobin (HGB) 12.2 g/dL; white blood cell (WBC) count 7.6 x10^3^/μL, with predominating neutrophils of (82.3%); platelet count 315 x10^3^/μL; and C-reactive protein (CRP) concentration 56 mg/L and erythrocyte sedimentation rate (ESR) of 85 mm/1 hour. Lastly, a repeated strep throat test was negative, and therefore, throat culture was ordered. The patient was started on 1 g oral augmentin (amoxicillin/clavulanic acid), and parents were instructed to report if fever persists given the use of prescribed antibiotics for more than 48 hours. 

**Figure 2:**
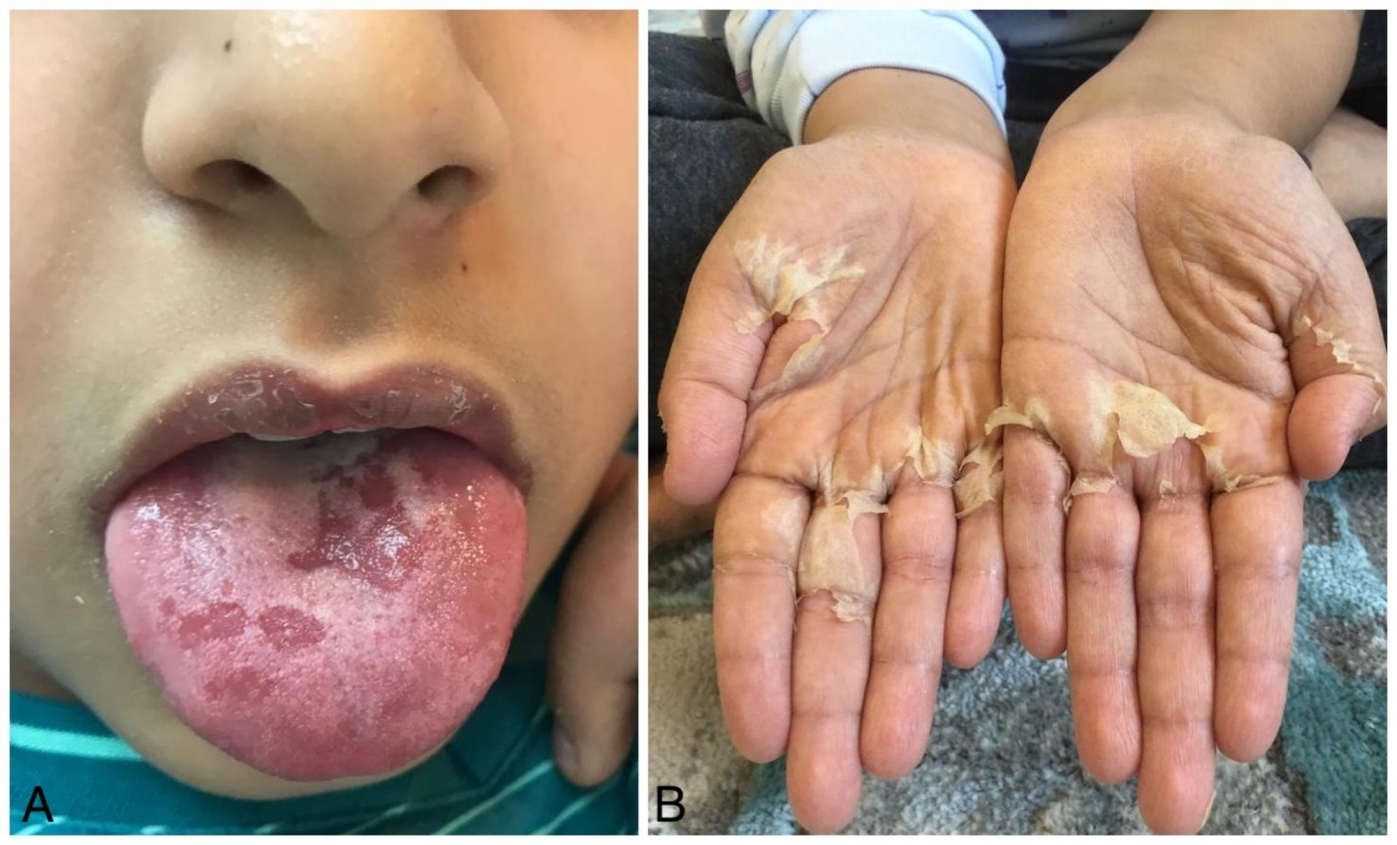
Symptoms observed during subsequent hospitalization Strawberry tongue (A); palmar desquamation involving both hands (B)

On the following day (day 4), the patient was admitted to the hospital ward due to persistent fever refractory to antibiotics. Physical examination was unremarkable for cervical lymph node enlargement suggesting resolution, whereas a neck ultrasound revealed corresponding unilateral hypo-echoic masses suggestive of cervical lymphadenitis, along with persistence of edema of hands and feet, and expansion of macular rash over his body. A few hours later during admission, the patient developed red eyes and characteristic early signs of strawberry tongue evident on physical examination (Figure 2A). Bacillus Calmette–Guérin (BCG) scar was not evident. Laboratory investigation at this time revealed elevated liver function test (LFT), alanine aminotransferase (ALT) of 186 IU/L, aspartate aminotransferase (AST) of 215 IU/L, gamma-glutamyltransferase (gamma-GT) of 180 IU/L, albumin serum of 28 g/L, serum protein of 78 g/L, and normal platelet count. Blood serology tests of Cytomegalovirus, measles, Epstein-Barr virus (EBV), and hepatitis A, B, and C reported a negative titer level. Urine test showed 10 cells/hpf of WBC with negative nitrite. Echocardiography was done and was normal. Given previous information, on day five (second day of admission), the patient was highly suspected of having typical KD based on clinical presentation consistent with poor outcome predictor (↑CRP, ↑LFT, ↓albumin) and was started on intravenous immunoglobulin (IVIG) 2 g/kg over 12 hours and a high aspirin dose of 80 mg/kg/d every six hours accordingly. The fever did not resolve within 36 hours, for which the patient was started on a second dose IVIG 2 g/kg over 12 hours. On day 10, the patient was noticed to have skin peeling involving both hands resembling sheet-like desquamation involving the palmar areas (Figure 2B). With that being said, after close observation for 48 hours, the patient was discharged home on a low-dose aspirin of 5 mg. Repeated echocardiography on day 14 was normal, and CBC follow-up showed platelet 609 x10^3^/μL consistent with thrombocytosis.

## Discussion

Despite the well-adapted clinical criteria for the diagnosis of childhood KD, previous reports have shown divergent presentations and a manifold array of clinical presentations. Few of the reports have demonstrated patients who yielded a final diagnosis of KD have initially presented with signs and symptoms of pancreatitis and severe shock, which all add to the complexity of diagnosing such illness [12-13]. Not to leave behind the adult variant of KD that was described by a case series, highlighting demographic variations [14]. This all brings to light the necessity of a high index of suspicion and clinicians’ knowledge of atypical presentations of KD to preserve patient’s health and proper management. 

This paper thus reported a 13-year-old male who initially presented with unilateral cervical lymphadenopathy and papular rash of upper extremities. Despite the fact that rash and lymphadenopathy are criterion symptoms of KD, the patient’s age and absence of other classical symptoms of KD initially have led us to consider other diagnoses including viral, group A beta-hemolytic streptococcal infection, and EBV which may also present similarly.

## Conclusions

The diagnosis of KD should be considered in children presenting with cervical lymphadenopathy and a maculopapular rash, especially those who are old and in the situation where antibiotics were shown ineffective. In such atypical cases, pivotal pediatric cardiologist consultation should be a priority to reach early and proper management with minimum morbidity. 
